# Computational drug repurposing in the age of COVID-19: mixing antiviral cocktails in silico

**DOI:** 10.1038/s41746-022-00599-5

**Published:** 2022-04-22

**Authors:** James A. Diao, Marium M. Raza, Kaushik P. Venkatesh, Joseph C. Kvedar

**Affiliations:** Harvard Medical School, Boston, MA USA

**Keywords:** Virtual screening, Viral infection

## Abstract

As clinicians and scientists gather more data on the clinical trajectory of COVID-19 and the biology of its causative agent, the SARS-CoV-2 virus, novel strategies are needed to integrate these data to inform new therapies. A recent study by Howell et al. introduces a network model of viral-host interactions to produce explainable and testable predictions for treatment effects. Their model was consistent with experimental data and recommended treatments, and one of its predicted drug combinations was validated through in vitro assays. These findings support the utility of computational strategies for leveraging the vast literature on COVID-19 to generate insights for drug repurposing.

The traditional drug development process takes around a decade to produce, validate, and approve each new medication. This protracted timeline is a nonstarter for responding to fast-moving viruses like SARS-CoV-2, which has already produced more than a dozen monitored variants and caused more than six million deaths from coronavirus disease (COVID-19)^1^. Instead, today’s COVID-19 treatment arsenal largely comprises drugs developed for other diseases, including the Ebola drug remdesivir and the rheumatoid arthritis drug tocilizumab.

Drug repurposing offers several advantages over discovery from scratch, including abundant safety data, accelerated approval timelines, and established manufacturing systems^2^. But with thousands of drugs to choose from, identification of the most promising candidates presents a formidable challenge. This problem compounds with investigation of drug combinations, which increases the search space by orders of magnitude and introduces the possibility of drug interactions. Using a systems modeling approach, Howell et al^3^. developed a network representation of host-viral interactions to rapidly predict combination drug effects against severe COVID-19.

Howell and colleagues built their model using BioModelAnalyzer (BMA)^4^, a freely available and open-source toolset developed by Microsoft Research for constructing and verifying qualitative network models. Originally used to study leukemia and breast cancer^5^, BMA was applied to COVID-19 by manually encoding knowledge on viral interactions with lung cells and the immune system as described in the literature. Proteins and processes for SARS-CoV-2 and its human host were represented as nodes with varying degrees of activity and joined by edges representing activating or inhibitory effects. To validate their model, Howell and colleagues compared model predictions with observed outcomes from 81 in vitro experiments, six common COVID-19 treatments, and three genetic risk factors. These tests were used to iteratively expand and refine the model until it reproduced over 90% of experimental observations. The resulting network aims to characterize our current understanding of the complex biological interactions underlying COVID-19.

Beyond its descriptive power, the model can also be “executed” to simulate how modifying one node would affect the others. For example, camostat is a drug that inhibits TMPRSS2, a surface protein involved in viral entry. Administration of camostat is represented by deactivation of the TMPRSS2 node. Through its outgoing connections, TMPRSS2 modifies its neighboring nodes “viral fusion” and “syncytia formation,” which in turn modify their respective neighbors, thus propagating an initial perturbation through the network. These cascading regulatory interactions may eventually converge into a stable state with numeric readouts for viral replication, immune infiltration, inflammation, cell death, and other phenotypes. By toggling nodes affected by relevant drugs and assessing the results, the authors are able to screen for new therapies. In total, Howell and colleagues assessed 74 medicines that are clinically approved or undergoing phase II/III trials, both individually and in pairs.

This combinatorial investigation yielded nine promising drug combinations. Several treatments were predicted to inhibit viral replication, which could prevent progression to severe disease. Others were predicted to decrease inflammation, which could reduce immune-mediated lung injury. Unlike many competing models, Howell et al.’s approach allows researchers to trace a drug candidate’s systems-level mechanism of action and quantify off-target effects. For example, rapamycin/ruxolitinib were predicted to effectively target inflammation but also increase viral entry. As a result, the authors note that subsequent studies could be designed toward late-stage treatment when viral entry has already peaked and inflammatory damage is of greater concern. For in vitro validation, the authors tested a favored drug candidate, camostat/apilimod, in cultured human cells that were then infected with SARS-CoV-2. Cells treated with both drugs expressed fewer viral proteins than cells treated with either drug alone, suggesting improved inhibition of viral entry and replication.

Several directions remain for future work. First, automated methods for model construction may improve scalability and adaptability to the rapidly expanding COVID-19 literature. In the meantime, researchers may use BMA’s no-code interface to update existing models with new information or redesign them for new variants. Second, investigation is needed to integrate competing strategies for drug repurposing and triangulate the most promising candidates^6^. For example, host targets identified by Howell et al. may be further investigated with structure-based screening of approved drugs^7^ or combined with data on drug toxicity, selectivity, stability, and clearance^8^. Third, higher-order drug combinations remain less explored. Future work may replace exhaustive evaluation with heuristic search algorithms to efficiently evaluate combinations of three or greater. Lastly, given the sizable failure rate of both repurposed and de novo treatments^9^, moving beyond in vitro validation to animal models and human trials will provide the ultimate test of efficacy for predicted therapies.

Computational drug repurposing has informed therapeutic development from the first days of the pandemic. As early as February 2020, researchers at BenevolentAI had proposed baricitinib, a rheumatoid arthritis medicine, for treating COVID-19^10^. After several randomized trials, baricitinib received FDA authorization and was incorporated into national and global care recommendations^11^. Many other crucial treatments for COVID-19, while not discovered computationally, were also repurposed. These stories offer hope that similar strategies may soon transform the care of other high-priority diseases, such as influenza, HIV, and many cancers. In an important step towards this goal, Howell and colleagues demonstrate that their network model developed for cancer could be credibly repurposed for COVID-19 to identify promising candidates hidden in plain sight.

## References

[1] WHO. WHO Coronavirus (COVID-19) dashboard. https://covid19.who.int/ (2019).

[2] Hernandez JJ (2017). Giving drugs a second chance: overcoming regulatory and financial hurdles in repurposing approved drugs as cancer therapeutics. Front. Oncol..

[3] Howell R (2022). Executable network of SARS-CoV-2-host interaction predicts drug combination treatments. NPJ Digit Med.

[4] Benque D (2012). BMA: Visual tool for modeling and analyzing biological networks. Comput. Aided Verif.

[5] Clarke MA, Fisher J (2020). Executable cancer models: successes and challenges. Nat. Rev. Cancer.

[6] Jin, W. et al. Deep learning identifies synergistic drug combinations for treating COVID-19. *Proc. Natl. Acad. Sci. USA*10.1073/pnas.2105070118 (2021). 10.1073/pnas.2105070118 PMC8488647 34526388

[7] Ahmed, M. et al. Identification of atovaquone and mebendazole as repurposeddrugs with antiviral activity against SARS-CoV-2 (Version 6). *ChemRxiv*10.26434/chemrxiv-2021-b3fv1-v7 (2021).

[8] Hughes JP, Rees S, Kalindjian SB, Philpott KL (2011). Principles of early drug discovery. Br. J. Pharmacol..

[9] Venkatesan P (2021). Repurposing drugs for treatment of COVID-19. Lancet Respir. Med.

[10] Richardson P (2020). Baricitinib as potential treatment for 2019-nCoV acute respiratory disease. Lancet.

[11] Kalil AC, Stebbing J (2021). Baricitinib: the first immunomodulatory treatment to reduce COVID-19 mortality in a placebo-controlled trial. Lancet. Respir. Med..

